# Establishing a standardized training program for robotic surgeons in China

**DOI:** 10.1097/JS9.0000000000003307

**Published:** 2025-08-22

**Authors:** Mingwei Zhang, Peiming Zhang, Yunzhang Cheng, Tianyi Zhang

**Affiliations:** a School of Health Sciences and Engineering, University of Shanghai for Science and Technology, Shanghai, China; b Lin-gang Medical Device Innovation Center, China (Shanghai) Pilot Free Trade Zone, Shanghai, China

*Dear Editor,*

Surgical robots for endoscopic applications are high-end medical devices designed to perform various complex minimally invasive surgeries, they have significant advantages such as minimally invasive procedures, enhanced precise, improved flexible, and tremor-free operation. With advancements in visual systems, surgical flexibility, and ergonomic design, robot-assisted surgery has gained increasing popularity in clinical applications across China. However, the integration of this novel technology into operating rooms introduces potential risks of human error and technical malfunctions, which may increase surgical risks for patients^[1]^. In the UK, 10–15% of surgical patients experienced adverse events, 50% of which were preventable^[2]^. Between 2000 and 2013, a total of approximately 10 624 adverse events related to robot-assisted surgery were reported across various surgical departments in the United States^[3]^. A global independent review of health technology hazards identifies insufficient robotic surgery training as one of the top ten risks faced by patients^[4]^.

Compared with conventional surgical instruments, surgical robots demonstrate higher operational specificity and technical complexity, requiring an extended learning curve. Substantial evidence indicates that comprehensive robotic surgery training significantly enhances procedural accuracy, operational efficiency, and risk assessment capabilities, while reducing operative time and patient recovery periods^[5]^. Consequently, rigorous and systematic training constitutes an essential prerequisite for clinical implementation of robot-assisted surgery. Surgical robots, as the Class III medical devices (China’s highest-risk category requiring strict regulation), have been strictly managed by China’s National Health Commission as national restricted technologies. Standardized training program and evaluation methods play crucial roles in ensuring surgical quality and patient safety through regulated clinical application.

While the Chinese surgical robot market remains dominated by the Da Vinci Surgical System, domestically developed systems from companies such as Tumai, Jingfeng, and KangDuo have achieved limited clinical deployment. Nevertheless, standardized training program remain underdeveloped, with no nationally unified curriculum and evaluation methods. Current training programs primarily originate from manufacturer-developed curriculum, resulting in significant variability in training content, duration, and formats across different systems.

To address these challenges, our research team has developed a modular, phased, and competency-based training framework through interdisciplinary collaboration with experts in surgery, biomedical engineering, healthcare administration, and regulatory affairs. This initiative proposed a standardized training program and novel scoring scale specifically designed for China’s robotic surgery ecosystem (Figs. 1 and 2).

**Figure 1.**
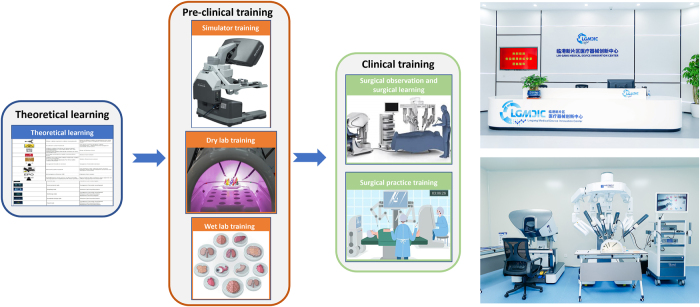
Schematic of the training program. The three-phase curriculum integrates theoretical learning, preclinical training, and clinical training, including simulator training, dry lab training, wet lab training, surgical observation and surgical practice training procedure.

**Figure 2.**
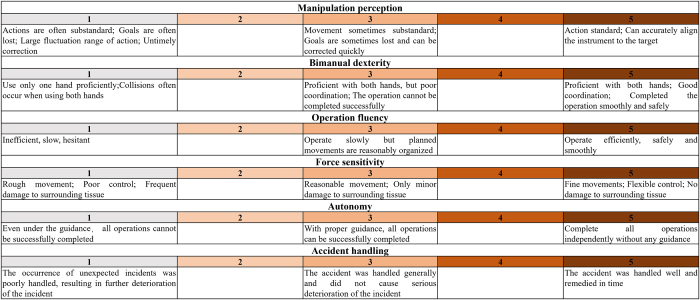
The novel scoring scale evaluates trainees’ robotic surgical competency across seven dimensions: manipulation perception, bimanual dexterity, operation fluency, force sensitivity, autonomy, accident handling. Operative performance is quantitatively assessed through consensus evaluations by five certified expert robotic surgeons.

This training program is expected to significantly enhance the safety profile and procedural standardization of robot-assisted surgery within China. The curriculum and scoring scale will undergo continuous refinement based on surgical robot technology updates, emergency incident analysis, and post-market clinical outcome monitoring. To facilitate the implementation of this training program, we have established a regional training hub within the Lin-gang Medical Device Innovation Center, serving as the primary implementation platform for this training program. Our article is compliant with the TITAN Guidelines 2025 – governing declaration and use of AI^[6]^.

## Data Availability

Not applicable.
